# Endocrine Adaptations to Prolonged Fasting: From Physiology, Clinical Uncertainties, Translational Challenges to Healthspan Implications

**DOI:** 10.3390/nu17243949

**Published:** 2025-12-17

**Authors:** Rok Herman, Jure Trsan, Luka Lipar, Mojca Jensterle, Andrej Janez

**Affiliations:** 1 Department of Internal Medicine, Faculty of Medicine, University of Ljubljana, 1000 Ljubljana, Slovenia; rok.herman@kclj.si (R.H.);; 2 Department of Endocrinology, Diabetes and Metabolic Diseases, University Medical Centre Ljubljana, 1000 Ljubljana, Slovenia; 3 Department of Cardiology, University Medical Centre Ljubljana, 1000 Ljubljana, Slovenia

**Keywords:** prolonged fasting, fasting, endocrine adaptations, metabolic switch, healthspan

## Abstract

Objectives: Intermittent fasting regimens that include periods of prolonged fasting may mimic certain well-documented benefits of calorie restriction. In this narrative review, we synthesize preclinical and human data on endocrine adaptations during prolonged fasting protocols. Methods: We conducted a structured search of relevant databases, followed by data extraction and synthesis, with a focus on endocrine adaptations during prolonged fasting and their potential implications for healthspan. Results: Across various endocrine axes, prolonged fasting appears to induce a reproducible pattern marked by diminished anabolic signaling and transient activation of potential stress resilience pathways. However, the evidence is limited by small sample sizes, short follow-up durations, methodological heterogeneity, and dependence on surrogate endpoints. Endocrine outcomes are frequently secondary and reported inconsistently. Potential risks include transient hypogonadism, relative hypothyroidism, hypercortisolemia, orthostatic intolerance, electrolyte imbalances, catabolic loss of lean mass, and refeeding challenges. Conclusions: Overall, prolonged fasting activates conserved endocrine mechanisms that may confer plausible cardiometabolic benefits; however, their translation to clinical practice remains speculative. We highlight key knowledge gaps and propose directions for future research in this emerging field.

## 1. Introduction

Intermittent fasting (IF) encompasses prespecified periods of almost complete or complete caloric abstinence, practiced for durations ranging from a few hours to several days or longer. Definitions vary; however, according to the 2024 international consensus, prolonged fasting denotes 4 or more consecutive days. Calorie restriction (CR) refers to a sustained reduction in energy intake below that required to maintain current body weight, without inducing malnutrition [1]. Even when total energy intake is matched, fasting—through discrete intervals of complete caloric abstinence—elicits physiologic adaptations that differ substantially from those induced by continuous CR.

Studies in overweight humans indicate that even short-term CR of 6 months can significantly improve several cardiovascular risk factors, insulin sensitivity, and mitochondrial function, irrespective of weight loss [2]. Recently, IF has gained prominence as a dietary pattern that some individuals find easier to sustain long-term than continuous CR and may confer superior metabolic effects [3,4]. Many of the benefits of IF, including effects that may surpass those achieved with a continuous CR, are hypothesized to be mediated by activation of the metabolic switch. It is believed that, via these mechanisms, fasting favorably modulates general health indicators and may influence the aging process and the development and progression of aging-related chronic diseases [4]. The metabolic switch denotes the transition from carbohydrate-dependent metabolism, mostly reliant on exogenous glucose and hepatic glycogen, to fatty acids and fatty acid-derived ketones [5]. Changes are characterized by increased adipose lipolysis, hepatic β-oxidation, and ketogenesis. Circulating ketone concentrations begin to rise after 8–12 h of fasting, whereas the metabolic switch typically occurs within 12–36 h, contingent on hepatic glycogen stores, insulin sensitivity, and physical activity. It leads to lower insulin and leptin concentrations, rising glucagon and catecholamines, and a progressive shift of the brain and peripheral tissues toward ketone utilization [6]. Ketone bodies are not merely energy substrates; they also modulate the expression and activity of numerous proteins and signaling molecules promoting autophagy and cellular stress resistance [7].

A major limitation of the existing literature is that the vast majority of human studies have been conducted using short-term fasting protocols, typically lasting between 12 and 48 h, or intermittent paradigms such as time-restricted eating, alternate-day fasting, or fasting-mimicking diets. These regimens do not recapitulate the physiological conditions of prolonged fasting, and most hallmark endocrine adaptations, as they emerge only after 48 to 72 h and intensify beyond the fourth day of energy deprivation. Consequently, conclusions drawn from shorter fasting protocols often mischaracterize or underestimate the true metabolic and hormonal phenotype of prolonged fasting and provide little insight into either the potential therapeutic benefits or the spectrum of risks that emerge only with sustained, multi-day energy deprivation. Short-term interventions capture only the earliest stages of substrate switching and acute counterregulatory responses, whereas prolonged fasting engages a qualitatively distinct, deeper endocrine and metabolic program. Without rigorous differentiation between these paradigms, findings from short-term or intermittent fasting studies risk being inappropriately generalized, obscuring the unique physiological profile, potential therapeutic value, and safety considerations that are specific to fasting durations of four days or more. Importantly, medically supervised prolonged fasting protocols lasting 4–21 days have now been systematically studied in some large human cohorts, providing foundational data on physiological adaptations, tolerability, and safety [8].

Nonetheless, IF elicits metabolic and cellular remodeling that continuous daily restriction cannot fully reproduce. Recurrent cycles of fasting and refeeding generate beneficial oscillations in core pathways—periods of ketosis, hypoinsulinemia, and autophagy followed by nutrient-driven rebuilding. Studies have demonstrated that IF has disease-modifying effects encompassing a wide range of chronic disorders, including obesity, diabetes, cardiovascular disease, cancers, and neurodegenerative brain diseases [2,4,9,10]. IF might modulate those states through weight loss and by potentially ameliorating insulin resistance, lowering blood pressure and resting heart rate, increasing heart rate variability, improving levels of low-density lipoprotein and triglycerides and reducing markers of systemic inflammation and oxidative stress that are associated with atherosclerosis [11,12,13].

Emerging human studies demonstrate that IF is associated with improved cognitive performance in older adults and in individuals at various stages of the Alzheimer’s disease continuum, alongside changes in systemic biomarkers consistent with enhanced autophagy, mitochondrial and sirtuin signaling, antioxidant capacity, and DNA damage–response programs [14,15,16]. Collectively, these findings support increased neuronal stress resistance in humans; however, evidence specific to Parkinson’s disease remains limited, and larger, longer randomized trials are needed to determine effects on disease trajectories [4,17,18]. In relapsing–remitting multiple sclerosis, prolonged fasting demonstrated neurofilament light chain levels reduction, depressive symptoms improvement and cardiometabolic benefits [19].

In oncology, fasting interventions are being evaluated in three principal formats, namely (i) short-term fasting, (ii) fasting-mimicking diets, and (iii) time-restricted eating, each intended to leverage the fasting metabolic switch. Early human studies and reviews suggest these approaches are feasible in carefully selected, non-cachectic patients and can remodel systemic aspects of antitumor immunity, with signals for improved treatment tolerance and patient-reported outcomes [20,21,22,23,24]. However, effects on disease control and survival remain inconclusive, with heterogeneity in protocols and endpoints limiting firm recommendations [24]. In practice, fasting interventions should be used, if at all, under protocolized supervision with nutrition monitoring and clear stopping rules, while larger, well-controlled trials standardize definitions, refine patient selection, and establish efficacy and safety across cancer types and therapies [25].

Although the fasting literature is extensive, most human research to date has focused narrowly on metabolic or weight-related outcomes, while rigorous evaluations of endocrine adaptations remain comparatively sparse and methodologically heterogeneous. Existing studies vary widely in fasting duration, dietary permissiveness, macronutrient context, refeeding protocols, participant selection, and the depth of hormonal characterization. Sample sizes are typically small, study populations highly selective, and protocols insufficiently standardized to allow meaningful comparison across trials or robust generalization to broader clinical populations. Furthermore, many studies rely on single time-point hormone measurements, lack pulsatility analyses, omit sex- and phenotype-specific stratification, and often do not account for menstrual-cycle phase, adiposity, baseline metabolic health or circadian endocrine variation, all of which critically modulate hormonal responses to nutrient deprivation. This heterogeneity in study designs—ranging from small preclinical animal models to limited short-term human trials—limits direct comparisons and generalizability. Because dietary patterns differ in timing, macronutrient composition, and energy flux, they perturb endocrine axes in distinct ways, including the hypothalamic–pituitary–somatotropic (HPS), hypothalamic–pituitary–gonadal (HPG), hypothalamic–pituitary–thyroid (HPT), and hypothalamic–pituitary–adrenal (HPA) axes; renin–angiotensin–aldosterone system (RAAS); and adipokine networks. Accordingly, their hormone-specific effects and trade-offs—including potential benefits and liabilities—warrant systematic, axis-by-axis investigation.

Consequently, the field still lacks a coherent understanding of axis-specific thresholds, temporal dynamics, compensatory trade-offs, and inter-axis interactions. The safety profile of prolonged fasting also remains insufficiently defined, particularly with respect to reproductive, thyroid, adrenal, and mineralocorticoid physiology, where vulnerabilities are likely to differ substantially across sex, age, metabolic phenotype, and baseline endocrine reserve. These gaps highlight the need for harmonized fasting protocols, deep endocrine phenotyping, and risk-stratified human studies capable of distinguishing adaptive metabolic reprogramming from maladaptive hormonal stress. Addressing these challenges will be essential for establishing evidence-based fasting regimens that maximize physiological benefits while minimizing endocrine, metabolic, and clinical risks across diverse populations.

It is essential to distinguish fasting-related endocrine responses that are conserved and physiologically adaptive from clinically significant adverse effects that may arise when these responses are prolonged, amplified, or occur in vulnerable individuals. Adaptive changes typically represent reversible energy-conservation or stress-resilience programs, whereas maladaptive consequences include persistent hypogonadism, electrolyte disturbances, orthostatic intolerance, hypercortisolemia, or refeeding complications. This distinction frames our evaluation of the evidence across endocrine systems.

**Figure 1:**
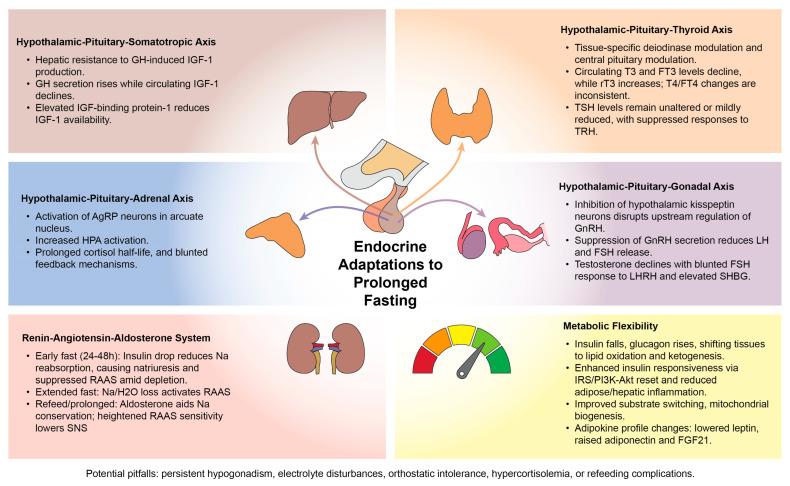
Endocrine adaptations to prolonged fasting, the mechanistic synthesis of the current literature. Legend: GH—growth hormone; IGF-1—insulin-like growth factor 1, AgRP—agouti-related peptide; HPA—hypothalamic–pituitary–adrenal; RAAS—renin–angiotensin–aldosterone; SNS—sympathetic nervous system; T3—triiodothyronine; FT3—free triiodothyronine; T4—thyroxine; FT4—free thyroxine; TSH—thyroid-stimulating hormone; TRH—thyrotropin-releasing hormone; GnRH—gonadotropin-releasing hormone; LH—luteinizing hormone; FSH—follicle-stimulating hormone; SHBG—sex-hormone binding globulin; FGF21—fibroblast growth factor 21.

We conducted a structured narrative review of the endocrine and metabolic effects of fasting. Searches were performed in PubMed and Google Scholar without date restrictions, limited to human and relevant preclinical studies published in English. The following search terms were used individually and in combination: “fasting”, “intermittent fasting”, “calorie restriction”, “prolonged fasting” paired with “IGF-1”, “growth hormone”, “somatotrophic axis”, “TSH”, “thyroid”, “thyroid axis”, “cortisol”, “adrenal axis”, “stress response”, “RAAS”, “aldosterone”, “renin-angiotensin-aldosterone”, “ketones”, “insulin”, “insulin sensitivity”, “insulin resistance”, “metabolism”, “metabolic switch”, “ketosis”, “metabolic flexibility”, “adipokines”, “leptin”, “ghrelin”. Reference lists of key articles and recent reviews were hand-searched to identify additional relevant studies. Given the heterogeneity of fasting protocols and endpoints, study selection prioritized mechanistic endocrine outcomes, controlled intervention designs, and clearly defined fasting exposures. No formal quality scoring was applied, but studies were evaluated for methodological clarity, consistency of outcome reporting, and relevance to endocrine pathways. The main findings of our review are summarized in Figure 1 and elaborated in detail in the subsequent axis-specific sections.

## 2. Endocrine Pathways in Prolonged Fasting

### 2.1. Hypothalamic–Pituitary–Somatotropic Axis

In recent decades, the HPS axis has attracted considerable attention in gerontological research, particularly regarding the effects of its activity and modulation on both healthspan and lifespan. Mounting evidence links subtle changes in its activity to the development of several age-associated conditions, including malignancies, neurodegenerative disorders, cardiovascular disease, and metabolic dysfunction [26].

Growth hormone (GH) is secreted by the anterior pituitary in discrete pulses, with approximately 70% of its daily output occurring during nocturnal bursts. Its release is primarily driven by growth hormone-releasing hormone (GHRH) and ghrelin, whereas somatostatin provides tonic inhibition [27]. Upon release, GH binds to its receptor, which is ubiquitously expressed across tissues, triggering several downstream signaling cascades, including JAK2-STAT5, MAPK-ERK1/2, PI3K-Akt-mTOR, and PLC/PKC/Ca^2+^ pathways. These signaling events culminate in enhanced transcription and hepatic synthesis of insulin-like growth factor-1 (IGF-1), which mediates many of GH’s anabolic effects and may also participate in metabolic adaptations to fasting [28]. GH and IGF-1 are pleiotropic hormones with important roles in fetal development, growth during childhood and adolescence, and adult tissue homeostasis. Through overlapping mechanisms, they promote the maintenance of lean and bone mass, support cellular differentiation and survival, and influence mitochondrial homeostasis [29]. Under conditions of nutrient abundance, GH acts synergistically with insulin to enhance amino acid transport and protein synthesis. In contrast, during fasting, it promotes lipolysis and protein sparing, facilitating a metabolic shift toward fat oxidation [29]. Excessive IGF-1 activity has been implicated as a risk factor for several cancers due to its strong mitogenic and anti-apoptotic activity, primarily mediated through regulation of the cell cycle, inhibition of apoptosis, and enhanced cell survival. These effects are largely driven by activation of insulin receptor substrates (IRS-1/2) and downstream PI3K–Akt–mTOR signaling [26,30,31,32].

Multiple animal models have demonstrated that mutations in genes regulating the GH/IGF-1/insulin signaling pathway can significantly increase lifespan across species ranging from nematodes to mammals [26,33,34]. For example, GH receptor knockout mice and IGF-1 receptor heterozygotes live significantly longer than wild-type controls [35,36]. Similar findings have been confirmed in multiple models of GH deficiency or resistance [37,38,39]. Beyond lifespan extension, these models also show reduced incidence of cancer and other age-related diseases [40]. Collectively, these findings support the concept that downregulation of GH/IGF-1 signaling is a conserved mechanism of healthy aging. Centenarians and their offspring often exhibit reduced circulating IGF-1 activity, and in some cohorts this correlates inversely with insulin sensitivity [41]. Familial cohorts enriched for exceptional longevity, such as those identified in the Long Life Family Study, have shown that circulating IGF-1 levels may serve as an informative age-related biomarker [42,43]. Genetic studies further support the link between reduced insulin/IGF-1 signaling and longevity. Variants in insulin/IGF-1 pathway genes have been associated with exceptional lifespan [44,45], and homozygosity for the GH receptor exon 3 deletion (d3-GHR) has been shown to become more prevalent with age and to extend life expectancy by roughly a decade [46]. A recent meta-analysis of 19 studies including over 30,000 individuals reported a U-shaped association between circulating IGF-1 and all-cause mortality, with both low and high IGF-1 levels conferring increased risk [47]. Furthermore, clinical data indicate that mortality is increased at both extremes of HPS axis dysfunction—in untreated acromegaly or growth hormone abuse, mainly due to increased rates of malignancies and cardiovascular death, but also in states of GH deficiency, primarily due to increased cardiovascular and cerebrovascular mortality associated with adverse body composition, dyslipidemia, insulin resistance, and endothelial dysfunction [48,49,50,51]. In addition, the mortality rates normalize after the achievement of the biochemical control of the underlying disease [52].

Human studies consistently demonstrate that prolonged fasting induces a paradoxical dissociation between GH and IGF-1. Within 3–5 days of fasting, circulating IGF-1 levels decline by up to 65%, while GH secretion rises due to loss of IGF-1–mediated negative feedback and reduced nutrient availability signaling [53,54,55,56]. This decline is amplified by increased levels of IGF-binding protein-1, which further reduces IGF-1 bioavailability and feedback inhibition [57]. Low insulin during fasting also contributes to hepatic GH resistance by downregulating GH receptor expression [58]. Shorter nutrient deprivation intervals also influence the axis: free and total IGF-1 fall progressively during acute fasting [59], while a 24 h water-only fast markedly increased GH secretion without reducing IGF-1, suggesting hypothalamic regulation independent of IGF-1 feedback [55]. CR alone yields less consistent endocrine responses. In the long term CALERIE trial increased IGFBP-1 was noted, without significant change in total IGF-1 after two years of intervention [60]. Pooled analyses suggest that IGF-1 reduction occurs primarily when CR is accompanied by protein restriction [61]. In a small open-label pilot trial involving 100 generally healthy participants (71 completing three cycles), a periodic 5-day fasting mimicking diet intervention, low in calories, sugars, and proteins but high in unsaturated fats, reduced fasting blood glucose by 11.3% and circulating IGF-1 by approximately 24%, while increasing IGFBP-1 by 1.5-fold. These changes were associated with improvements in risk factors related to aging, diabetes, cardiovascular disease, and cancer. Notably, glucose and IGF-1 remained lower than baseline (by ~6% and ~15%, respectively) even 5–7 days after resuming a regular diet following the last cycle [62]. Supporting this, a 10-day fast in healthy men decreased IGF-1 while increasing IGF-BP1, reducing bioavailability and aligning with downregulated signaling for potential mitogenic reduction, with protein sparing evident after day 5 [63]. More recently, an 8-day water-only fast in humans significantly decreased circulating IGF-1 and IGF-2 while markedly increasing GH [64].

In conclusion, these data suggest that prolonged fasting may induce a conserved downregulation of IGF-1 signaling despite elevated GH secretion, potentially mirroring longevity models; however, translation of this approach as a strategy to reduce mitogenic and anabolic activity associated with age-related diseases remains hypothetical at present. Energy intake and macronutrient composition play a critical role in modulating HPS axis activity. Future studies are needed to define optimal fasting durations and dietary contexts that maximize these potential benefits across diverse populations and metabolic phenotypes.

### 2.2. Hypothalamic–Pituitary–Gonadal Axis

Prolonged fasting exerts significant modulatory effects on the HPG axis, primarily through mechanisms that prioritize survival over reproduction during periods of nutrient deprivation. There is a rapid suppression of gonadotropin-releasing hormone (GnRH) secretion from the hypothalamus, resulting in reduced pulsatile release of luteinizing hormone (LH) and follicle-stimulating hormone (FSH) from the anterior pituitary. Key metabolic signals underlying these changes include reduced leptin and insulin levels, which act as peripheral indicators of energy status and inhibit hypothalamic kisspeptin neurons, a critical upstream regulator of GnRH release [65,66,67]. In various animal models Kiss1 mRNA and gonadotropin secretion are reduced during prolonged fasting [68,69]. Additionally, fasting-associated elevations in glucocorticoids may further lower HPG axis activity by exerting inhibitory effects at multiple levels [70].

In men, human studies on prolonged fasting consistently demonstrate suppressive effects on the HPG axis, characterized by reductions in FSH, LH, and downstream androgens; however, responses vary by age, duration, and baseline clinical state. For instance, a 48 h fast in healthy young men (small observational study of 9 participants) significantly lowered mean LH and FSH concentrations, pulse frequency, and testosterone levels, without altering LH pulse amplitude or cortisol levels, indicating early hypothalamic inhibition of reproductive function [71]. Similarly, a 3.5-day fast in young men suppressed pulsatile LH secretion by reducing burst frequency and mass, alongside decreased free testosterone and increased cortisol, while older men showed blunted responses, highlighting age-dependent effects [72]. In obese men undergoing a 10-day fast, serum FSH and testosterone declined significantly, with blunted FSH responses to luteinizing hormone-releasing hormone (LHRH) and increased urinary excretion of LH and FSH, suggesting altered pituitary responsiveness and potential changes in renal clearance [73]. An 8-day water-only fast in middle-aged healthy men reduced FSH, prolactin, total and free testosterone, and dehydroepiandrosterone (DHEA) by 17–36%, while elevating sex hormone-binding globulin (SHBG) without affecting LH, accompanied by decreased testicular and prostate volumes and improved lower urinary tract symptoms, possibly linked to fasting-induced ketosis and dehydration impacting the HPG axis [74]. In addition, a 5-day fast in normal-weight men decreased LH secretion rates and testosterone by 30–50% via reduced GnRH output, as evidenced by preserved pituitary responsiveness and reversal with pulsatile GnRH administration, underscoring hypothalamic GnRH suppression as a central mechanism [75]. Furthermore, in men with impaired sperm quality, a fasting mimicking diet (three 5-day cycles over 4 months) yielded trends toward improved progressive motility and concentration compared to controls, with reduced round cells, implying adaptive HPG responses without reported adverse effects [76].

Human studies in women examining prolonged fasting or fasting-mimetic approaches indicate subtle and generally protective effects on the HPG axis. These include temporary reductions in gonadotropin pulsatility, which vary according to menstrual cycle phase, body composition, and factors such as energy availability or polycystic ovary syndrome (PCOS), while generally preserving overall reproductive function. For instance, a 72 h fast in eumenorrheic women during the midfollicular or luteal phase did not alter LH pulsatility, FSH, estradiol, or subsequent progesterone levels, indicating resilience of reproductive function to short-term energy deprivation [77]. Similarly, a 3-day fast in normal-weight sedentary women during the midfollicular phase reduced LH pulse frequency and did not impact LH level, LH pulse amplitude, follicular development, or ovulation. Those changes suggest that acute fasting affects HPG axis activity but not cycle integrity [78]. In a small open-label prospective study of 8 women with hypothalamic amenorrhea (HA) due to chronic negative energy balance, recombinant leptin replacement over 2–3 months restored LH pulsatility, increased estradiol, and induced ovulation in a good third of participants, highlighting hypoleptinemia as a key mediator of HPG axis disruption in prolonged undernutrition [79]. A randomized trial of metreleptin in HA women over 36 weeks recovered menstruation in 70% and ovulation in over 50%, with rises in estradiol and progesterone, underscoring leptin’s role in reversing fasting-induced reproductive suppression [80]. For PCOS patients, a systematic review of IF protocols (time-restricted eating included) showed improvements in menstrual regularity (33–40%), reduced testosterone (9%), free androgen index (26%), and anti-Müllerian hormone, alongside elevated SHBG, without consistent changes in LH/FSH [81].

The longevity implications of the suppression of the HPG axis during prolonged fasting stem from evolutionary trade-offs in resource allocation during energy scarcity. In animal models, attenuation of gonadal hormone signaling activates conserved longevity-associated pathways (e.g., FOXO/DAF-16), leading to extended lifespan and delayed onset of pathologies like neurodegeneration and cancer [82]. In humans, epidemiological and clinical evidence suggests that lower lifetime exposure to sex steroids correlates with reduced incidence of hormone-dependent cancers (e.g., prostate in men and breast/ovarian in women) and improved metabolic outcomes, implying that reversible HPG axis downregulation induced by prolonged fasting could mimic these benefits without irreversible fertility loss [83,84]. Sex- and age-specific differences are evident across populations. In states of androgen excess, such as PCOS, this shift can attenuate hyperandrogenemia and improve menstrual cyclicity and ovulatory function, with concurrent gains in insulin sensitivity [81]. In men, reversible reductions in testosterone may mitigate androgen-driven cardiovascular risks and benign prostatic hyperplasia, and briefly dampen proliferative signaling, while leptin–kisspeptin coupling ensures rapid recovery of reproductive function during food reentry [78,80]. Nonetheless, potential drawbacks include exacerbated estrogen deficiency in postmenopausal women, accelerating menopause-associated bone loss or cognitive decline, underscoring the need for personalized approaches. Furthermore, there are substantial observational and epidemiological data linking hypogonadism in men to elevated CV risk, including increased incidence of atherosclerosis, coronary artery disease, CV events, and mortality [85,86].

Conceptually aligned with life-history (“disposable soma”) theory, periodic HPG axis downregulation reallocates resources from reproduction to somatic maintenance. The most prominent effect is a time-limited, reversible maintenance state with the net effect of slowing biological hallmarks of aging. However, prolonged or frequent cycles introduce the risk of sustained hypogonadism, bone loss, or fertility impairment [87,88].

### 2.3. Hypothalamic–Pituitary–Thyroid Axis

Thyroid hormones, primarily thyroxine (T4) and its biologically active metabolite triiodothyronine (T3), are key determinants of basal metabolic rate. Their synthesis and secretion are under the control of the HPT axis [89]. Prolonged fasting elicits marked adaptations of this axis, representing an evolutionarily conserved response aimed at conserving energy through downregulation of metabolic rate in the face of nutrient scarcity.

During extended fasting periods, circulating T3 and free T3 (FT3) levels typically decline, while the inactive metabolite reverse T3 (rT3) increases [90]. For example, in a human study of eight healthy subjects during the final 24 h of a 60 h fast, plasma T3 decreased from 1.73 ± 0.06 to 1.36 ± 0.04 nmol/L, while rT3 increased from 0.30 ± 0.06 to 0.44 ± 0.09 nmol/L [91]. In another human study involving 58 healthy subjects, after 24 h of fasting, FT3 decreased by 6% and rT3 increased by 16%, with more pronounced shifts at later time points [92]. Changes in T4 and FT4 are less consistent, showing slight decreases or stability. In the previously mentioned 60 h fasting study, plasma T4 remained unchanged at 103 ± 9 nmol/L (control) vs. 103 ± 10 nmol/L (fasting), with similar stability in FT4 (14.7 ± 1.0 vs. 16.1 ± 1.3 pmol/L). TSH levels remain unaltered or mildly reduced, deviating from the expected negative feedback response—for instance, mean 24 h TSH concentrations fell from 2.0 ± 0.3 to 1.0 ± 0.2 mU/L (*p* < 0.005) after 60 h of fasting, with no significant change in pulse frequency but reduced amplitude [91]. In addition, a 72 h fast in 17 healthy subjects resulted in a mild but significant decrease in TSH levels (from 1.1 ± 0.3 to 0.8 ± 0.1 mU/L by day 3, *p* < 0.05) [93]. This pattern is observed across human and rodent studies, with fasting leading to suppressed TSH responses to TRH stimulation and decreased TSHβ mRNA expression in the pituitary [94,95]. Human evidence from prolonged TRH infusions during fasting (up to 72 h) demonstrates blunted TSH responses, with TSH increase being reduced by approximately 50% compared to fed states, as was already reported in undernutrition models [96].

These adaptations are most likely mediated by a combination of tissue-specific regulation of deiodinase enzymes activity and central modulation of pituitary function. Peripherally, fasting lowers type I and type II iodothyronine deiodinase activity in the liver, shifting T4 metabolism toward inactivation and reducing T3 production, while elevating type III iodothyronine deiodinase to enhance rT3 conversion [90]. Centrally, hypothalamic tanycytes upregulate type II iodothyronine deiodinase expression to maintain local T3 concentrations, thereby limiting local signal for TRH release [90]. Declining leptin levels during fasting further modulate this process by influencing hepatic type III iodothyronine deiodinase, and pathways such as mTOR and constitutive androstane receptor contribute to deiodinase shifts [97]. Thyroid hormone transporters like monocarboxylate transporter 8 (MCT8) and MCT10 exhibit variable responses, with upregulation in some tissues, like white adipose tissue, to facilitate localized thyroid hormone bioavailability [90]. Additionally, the unchanged TSH pulse frequency during fasting suggests that the intrinsic rhythm of pulsatile TSH secretion remains stable, while the decrease in pulse amplitude points to modulated pituitary responsiveness, potentially involving increased somatostatin inhibition, altered T3 receptor occupancy within the pituitary, or direct effects of fasting on thyrotrophs independent of hypothalamic factors [98,99,100].

Such processes induce a hypometabolic state, as indicated by a 20–30% decline in resting metabolic rate and a transition to ketone-based metabolism between days 3 and 6 of fasting, thereby facilitating energy conservation under stringent conditions [90,98]. Regarding potential longevity outcomes, the hypothyroid-like profile triggered by fasting echoes patterns seen in CR, wherein persistent declines in T3 concentrations without significant changes in T4 or TSH have been hypothesized to correlate with lifespan extension in rodents, nonhuman primates, and possibly humans, though human evidence remains limited [101,102,103]. For example, long-term CR in humans lowers T3 by approximately 20%, independent of body fat changes, and is hypothesized to prolong healthspan by diminishing metabolic rate, oxidative stress, and cellular senescence [104,105]. Inverse associations between TSH levels and lifespan in centenarians and long-lived mutants support this, suggesting that moderated thyroid activity mitigates free-radical production and age-related inflammation [106].

Overall, alterations in thyroid hormone levels during extended fasting converge with broader endocrine adjustments, which may promote metabolic adaptability and protect against mechanisms of aging, although human studies on prolonged fasting intervals are sparse and require circumspect evaluation for clinical applications [102].

### 2.4. Hypothalamic–Pituitary–Adrenal Axis

Prolonged fasting elicits significant changes in HPA axis function, primarily through increased activation in response to the metabolic stress of energy deprivation, leading to increased adrenal glucocorticoid secretion. This activation manifests as elevated cortisol levels, which facilitate gluconeogenesis, lipolysis, and overall metabolic flexibility to sustain homeostasis during nutrient scarcity. Recently, this well-known response has gained further mechanistic clarity. Fasting-activated agouti-related peptide (AgRP)-expressing neurons in the arcuate nucleus project to the paraventricular nucleus of the hypothalamus and presynaptically disinhibit corticotropin-releasing hormone neurons via GABAergic afferents, thereby initiating the HPA cascade [107]. Multiple human and animal studies confirm that cortisol rises early during fasting and stays elevated during prolonged fasts. For instance, in a recent study involving 8 days of water-only fasting in middle-aged men, serum cortisol concentrations increased more than twofold [64]. Another study similarly showed increased cortisol secretion combined with a shift in diurnal cortisol secretion pattern [108]. A systematic review and meta-analysis of studies in which cortisol was measured following CR showed that acute fasting exerts a strong cortisol-raising effect and that serum cortisol levels are negatively associated with the duration of CR [109]. Supporting this signal, in a controlled 10-day fast with physical activity, urinary free cortisol decreased by 36% by day 10 and remained low during refeeding [63].

During short-term fasting, cortisol rises acutely to support survival, while in chronic starvation, sustained hypercortisolemia persists, driven by central HPA activation, prolonged cortisol half-life, and blunted feedback mechanisms. Metabolically elevated cortisol promotes gluconeogenesis, promotes amino acid and lipid catabolism, and restrains peripheral glucose uptake, to uphold metabolic equilibrium for necessary glucose supply to critical tissues [97]. Activation during periodic fasting could exert longevity-promoting effects of reduced inflammation and cellular repair signaling, whereas during chronic conditions such as starvation, numerous negative long-term effects may prevail, like reduced bone and muscle mass [110,111]. One intriguing possibility is that activation of the HPA axis during prolonged fasting may paradoxically contribute to healthspan-promoting effects via reduction in low-grade inflammation and enhancement of repair signaling. Glucocorticoids are among the most potent endogenous regulators of inflammation and are pharmacologically exploited for their immunosuppressive effects [112]. In innate immune cells such as macrophages, glucocorticoid signaling can bias polarization toward more tissue-repair or M2-like phenotypes and dampen inflammatory activation cascades [113].

Moreover, suppressing chronic low-grade inflammation is widely recognized as a mechanism for slowing specific aspects of biological aging (so-called “inflammaging”), protecting tissues such as endothelium, brain, and kidney from cumulative oxidative and immune stress. In the fasting context, some clinical and preclinical studies have documented reductions in circulating inflammatory markers such as CRP, TNF-α, and IL-6 under intermittent or periodic fasting regimens, although these findings primarily reflect shorter fasting or dietary interventions [114]. In addition, glucocorticoid signaling may facilitate tissue homeostasis and repair. Indeed, cross-talk exists between glucocorticoid receptor signaling and key repair or stress-resistance pathways [115]. Furthermore, prolonged fasting could induce HPA axis adaptations such as changes in feedback sensitivity, glucocorticoid receptor expression and circadian rhythm and amplitude, but longitudinal human data are scant [116].

In general, the long-term implications of fasting-induced glucocorticoid elevation on peripheral tissues (e.g., muscle, bone, hippocampus) in the context of aging, catabolism, or stress tolerance remain incompletely defined. Furthermore, interindividual variation may modulate cortisol responsiveness to fasting stimuli, but systematic mechanistic comparisons are lacking. Finally, in special populations (e.g., adrenal insufficiency), fasting may pose risks of adrenal crisis [117].

### 2.5. Renin–Angiotensin–Aldosterone System

The RAAS is a key endocrine pathway regulating blood pressure, electrolyte balance, and fluid homeostasis. Its chronic dysregulation contributes to the pathogenesis of age-related disorders, including hypertension, cardiovascular disease, and metabolic syndrome, thereby indirectly accelerating biological aging [118,119]. Prolonged fasting produces a characteristic, time-dependent remodeling of the RAAS. In the early fast (24–48 h), insulin falls, and proximal tubular sodium reabsorption drops, resulting in a transient natriuresis with dissociation or relative suppression of RAAS signaling activity despite ongoing sodium and water depletion [120,121]. As fasting extends, negative sodium and water balance accrues, compensatory RAAS activation emerges, with rising plasma renin activity and variable aldosterone responsiveness [122,123]. Upon refeeding, aldosterone-dependent anti natriuresis is prominent and can be blunted by mineralocorticoid blockade, underscoring aldosterone’s role in post-fast sodium conservation [124]. Prolonged fasts appear to foster heightened RAAS sensitivity, resulting in adaptive downregulation of sympathetic nervous system activity and pronounced antihypertensive effects that may bolster vascular integrity and endothelial function [125]. In an early study, 68 borderline hypertensive patients underwent supervised water-only fasting (average 13.6 days) followed by low-fat, low-sodium vegan refeeding, with 82% achieving blood pressure ≤120/80 mm Hg and a mean reduction of 20/7 mmHg, especially in those with higher baselines. In a larger prospective observational study of 174 hypertensive patients undergoing medically supervised water-only fasting (average 10–11 days) preceded by a fruit-and-vegetable diet and followed by a low-fat, low-sodium vegan refeeding period, nearly 90% achieved blood pressure below 140/90 mmHg, with an average reduction of 37/13 mmHg and the most pronounced effects (60/17 mmHg) in those with stage 3 hypertension; all participants on antihypertensive medications discontinued them successfully [126]. While endocrine mechanisms were not directly examined in these early studies, the antihypertensive effects were presumed to be mediated through RAAS modulation via natriuresis and fluid shifts. This interpretation aligns with subsequent mechanistic research and supports a potential role for prolonged fasting in reducing age-related hypertension risk and enhancing healthspan [127]. In a prospective study of overweight and obese non-diabetic adults undergoing medically supervised prolonged water-only fasting (median 17 days) followed by whole-plant-food refeeding, significant reductions in systolic blood pressure were observed at the end of fasting, with these antihypertensive effects persisting post-refeeding with enduring benefits observed during refeeding such as lowered low-density lipoprotein (LDL) cholesterol and high-sensitivity *C*-reactive protein (hsCRP) levels, thereby implying reductions in cardiometabolic risk factors and systemic inflammation conducive to extended healthspan [128]. In a six-week follow-up to a prospective study involving overweight and obese non-diabetic adults who underwent medically supervised water-only fasting (median 14 days) followed by whole-plant-food refeeding, sustained reductions were observed in systolic blood pressure (−7.68 mmHg), diastolic blood pressure (−2.44 mmHg), body weight, lipids (total cholesterol and LDL), and inflammation (hsCRP), with transient increases in triglycerides and insulin resistance during refeeding resolving to baseline levels [129]. In the largest observational study to date, 1610 subjects undergoing supervised prolonged fasting (mean 10 days) experienced significant blood pressure reductions (mean −6.5/−3.8 mmHg overall; up to −24.7/−13.1 mmHg in severe hypertensives). Notably, 23.6% of medicated hypertensives discontinued antihypertensive drugs, with reductions in 43.5%, highlighting adaptive RAAS remodeling that persists into refeeding [130].

Although direct evidence that prolonged fasting modulates the RAAS to alter blood pressure remains limited, direct measurements from human IF studies provide important insights. In a cohort of controlled hypertensive patients undergoing Ramadan IF (approximately 16–18 h daily for 30 days), serum angiotensin II (Ang-II) levels and angiotensin-converting enzyme (ACE) activity significantly decreased post-fasting, while angiotensin I (Ang-I) levels increased, correlating with substantial reductions in 24 h systolic and diastolic blood pressure values. These RAAS alterations were predictive of blood pressure improvements, potentially mediated by suppression of vasoconstrictor pathways and enhancement of parasympathetic autonomic tone, as indicated by increased heart rate variability markers, suggesting cardiovascular benefits, including reduced sympathetic overdrive and hypertension risk [131]. Preclinical models offer a mechanistic framework: In aged Wistar rats, every-other-day fasting resets renal RAAS signaling by lowering plasma Ang II, elevating protective ACE2 and AT2R expression, and decreasing the AT1aR/AT2R ratio, thereby reversing age-related hypertension and increasing expression of the anti-aging protein klotho, which may integrate with sirtuins, PPARs, and AMPK pathways to mitigate oxidative stress, enhance renal function, and promote longevity [132]. Similarly, in mice on high-fat or high-fructose diets, IF shifts cardiac RAAS toward the vasodilatory ACE2/MAS axis, reducing left ventricular hypertrophy and improving metabolic parameters like blood pressure and lipids [133]. In preclinical rodent models, fasting elicits a tissue-specific downregulation of angiotensinogen in adipose tissue, with adipocyte angiotensinogen mRNA expression plummeting to about 14.6% of control levels and secretion dropping to 33% after 3 days in Sprague Dawley rats, while liver mRNA and serum concentrations remain unchanged. Following refeeding, these metrics rebound markedly, with mRNA rising to 228% and secretion to 183% of baseline, indicating a nutritionally responsive regulatory mechanism that could influence local adipose blood flow and fatty acid release [134].

In summary, prolonged fasting induces a dynamic, adaptive remodeling of the RAAS that transitions from initial suppression to compensatory activation, potentially fostering antihypertensive and anti-inflammatory effects that may hypothetically mitigate age-related cardiometabolic decline and enhance healthspan, pending further clinical validation in humans. These mechanisms, supported by human IF data and preclinical models, highlight RAAS modulation as a promising non-pharmacological pathway for promoting longevity, though further longitudinal studies in humans are needed to confirm direct causal links.

### 2.6. Insulin Sensitivity, Metabolic Flexibility, and Adipokine Modulation

Prolonged fasting elicits profound alterations in insulin dynamics, metabolic substrate utilization, and adipokine profiles. Metabolic flexibility is the ability of tissues to respond or adapt to conditional changes in metabolic demand by dynamically switching between substrates [135]. In the context of prolonged fasting, metabolic flexibility is forced by low substrate availability. As previously noted, during a fast, insulin concentration falls, glucagon rises, and peripheral tissues shift toward increased lipid oxidation and ketogenesis [136]. Within 12–24 h, hepatic glycogen depletion prompts a transition to gluconeogenesis and ketogenesis, with ketone bodies (e.g., β-hydroxybutyrate) rising to 1–2 mM by 48 h in humans, sparing glucose for obligate glucose-dependent tissues like the brain. This shift is mediated by counter-regulatory hormones such as glucagon, epinephrine, and cortisol, which activate lipolysis via hormone-sensitive lipase, releasing free fatty acids for β-oxidation [137,138]. Evidence from large, supervised cohorts demonstrates that prolonged fasting induces a stable and sustained ketogenic state beyond day 3–4, without progression to ketoacidosis or clinically relevant metabolic derangement, even in older individuals and those with cardiometabolic risk factors, provided medical supervision is ensured [139]. Upon refeeding, tissues exhibit enhanced insulin responsiveness, reflecting a physiological resetting of insulin signaling pathways [140,141]. Mechanistically, this improvement is mediated through decreased ectopic lipid deposition, restoration of insulin receptor substrate (IRS) and PI3K–Akt signaling, and attenuation of pro-inflammatory signaling in adipose and hepatic tissue [5]. In humans, prolonged fasting or IF improves HOMA-IR and postprandial insulin sensitivity independent of weight loss [12,142]. A case of repeated prolonged fasting (11–20 days) in type 2 diabetes patient demonstrated enhanced insulin sensitivity, with insulin decreasing from 14 to 4 mIU/L and HOMA-IR from 3.6 to 0.9 during fasting (overall 4.7 to 2.6), alongside glucose reductions and gut microbiota shifts resilient post-refeed [143].

In obese individuals, prolonged fasting (48 h) reveals metabolic inflexibility, with blunted shifts from glucose to lipid oxidation, associated with lower skeletal muscle mitochondrial respiratory chain content and reduced AMPK activity [144]. Over repeated fast–refeed cycles, adaptability can improve, meaning tissues become more responsive to substrate cues. For example, in mice fed a high-fat diet, maintaining a normal feeding pattern via 8 h daily fasting prevents impairment, as indicated by prompt decreases in respiratory exchange ratio during food deprivation. Human studies corroborate this, showing that 36 h fasting alters metabolomic profiles, with non-obese individuals exhibiting greater glucose variations but improved overall substrate switching compared to obese counterparts [145,146]. These enhancements in metabolic flexibility during prolonged fasting contribute to healthspan by reducing oxidative stress and inflammation, key drivers of aging. Activation of AMPK and PGC-1α during fasting promotes mitochondrial biogenesis and fatty acid oxidation, mirroring effects observed in caloric restriction that extend lifespan in rodents. In neurodegenerative models, ketone utilization protects neurons, improving cognitive function and motor performance, suggesting broader implications for age-related decline [137,147,148]. A remarkable case of repeated prolonged fasting (21 days/year for 45 years) demonstrated sustained metabolic flexibility, with cyclic variations in body weight and clinical parameters resolving post-refeeding, potentially reflecting adaptive insulin dynamics and ketogenesis that preserved healthspan into advanced age [149].

Adipose tissue acts as both a target and an effector of these metabolic adaptations. Adipokines undergo significant modulation during prolonged fasting, influencing insulin sensitivity and inflammation. Fasting decreases leptin and increases adiponectin concentrations, reflecting an endocrine shift toward insulin sensitization and lipid mobilization [150,151]. The fasting-induced decline in leptin, mediated by sympathetic activation and decreased energy availability, reduces hypothalamic inflammation and restores central insulin and leptin sensitivity [151]. Elevated adiponectin, particularly its high-molecular-weight form, enhances skeletal muscle fatty acid oxidation via AMPK activation, promotes hepatic insulin sensitivity, and exerts anti-inflammatory effects through suppression of NF-κB signaling [152,153,154]. These changes collectively improve metabolic efficiency and reduce risk factors associated with metabolic syndrome and age-related insulin resistance. A post hoc analysis of data from 1422 subjects demonstrated metabolic flexibility’s anti-inflammatory role through reduced ESR and CRP in elevated baselines, with ketone levels correlating to CRP changes, potentially via reduced insulin and enhanced adiponectin signaling [155]. Moreover, fasting may beneficially modulate novel adipokines such as omentin, apelin, and FGF21, all implicated in metabolic homeostasis and longevity [156,157,158]. FGF21, upregulated during prolonged fasting via PPARα activation, acts as a systemic signal of nutrient deprivation and energy deficit, promoting ketogenesis, lipolysis, and glucose uptake in an insulin-independent manner [159]. Chronic elevation of FGF21 is associated with lifespan extension in rodents, suggesting that fasting-induced intermittent activation of this axis may mimic some caloric restriction benefits [160,161]. In addition, a study of 10-day fasting in healthy adults demonstrated metabolic flexibility through fat mobilization, and excretion of bioaccumulated endocrine disruptors like arsenic, potentially linked to adipokine modulation and reduced inflammation [162].

## 3. Discussion

Prolonged fasting activates a coordinated network of endocrine adaptations that may promote metabolic efficiency, cellular maintenance, and resilience against age-related disease, though clinical translation requires further evidence [2,4]. Across endocrine axes, fasting induces a reproducible endocrine signature characterized by reduced anabolic drive and transient activation of stress resilience programs. These adaptations may become clinically maladaptive when they are prolonged, amplified, or imposed on vulnerable phenotypes as the same protective mechanisms that promote short-term survival and metabolic efficiency can transition into clinically significant adverse effects if fasting extends beyond physiological tolerance, leading to persistent hormonal imbalances, tissue damage, or functional decline [163,164,165].

Over the past decade, fasting interventions have been extensively investigated in relation to metabolic and cardiovascular outcomes, yet the endocrine dimension of these adaptations remains comparatively underexplored in this context. Current evidence on hormone-specific responses is fragmented, originating largely from small or short-term human studies and complemented by mechanistic insights from animal models, with few long-term, well-controlled clinical trials available to validate these findings. Consequently, most insights derive from small-sample human studies or animal data, underscoring the need for larger RCTs to distinguish robust evidence from preliminary observations. This gap is particularly consequential because fasting influences multiple endocrine axes regulating energy balance, stress adaptation, and reproductive function, while its use is rapidly expanding in the general population and increasingly considered for clinical and preventive applications. Understanding how these endocrine systems respond in individuals with established or subclinical endocrine disorders, including thyroid dysfunction, adrenal insufficiency, diabetes, and reproductive hormone imbalances, is critical to prevent adverse effects and to guide the safe and evidence-based implementation of fasting protocols in both clinical and preventive settings.

Current evidence is sufficient to support several cautious inferences about the endocrine responses to prolonged fasting. Suppression of GH/IGF-1 signaling decreases mitogenic and pro-aging signaling, mimicking longevity phenotypes observed in model organisms [34,35,166]. Periodic downregulation of the HPG and HPT axes reallocates resources from reproduction and thermogenesis toward somatic maintenance [82,87,101], while activation of the HPA axis and the release of FGF21 support substrate mobilization, autophagy, and tissue repair [107,112,159]. Remodeling of the RAAS and the adipokine milieu contributes to improved vascular tone, insulin sensitivity, and anti-inflammatory balance [118,127,150]. Collectively, these endocrine and metabolic shifts result in lower blood pressure, improved lipid and glucose homeostasis, enhanced mitochondrial efficiency, and potentially reduced risk of cardiometabolic, neurodegenerative, and neoplastic diseases [11,12,137]. Large cohorts confirm the safety and adaptive benefits of prolonged fasting; in 1422 subjects, including those with hypertension or type 2 diabetes, prolonged fasting enhanced physical and emotional well-being, and improved cardiometabolic risk factors, with adverse effects in <1% under supervision [167].

Endocrine adaptations to fasting may become clinically maladaptive when they are prolonged, amplified, or imposed on vulnerable patient phenotypes. Sustained suppression of the HPG axis can aggravate pre-existing estrogen deficiency, thereby accelerating bone loss and potentially worsening neurocognitive aging trajectories in postmenopausal women [168,169]. In men, repeated fasting-induced hypogonadism is typically reversible, but chronically depressed testosterone and gonadotropin secretion have been linked to adverse cardiometabolic profiles, endothelial dysfunction, and higher cardiovascular risk [170,171]. Persistent downregulation of thyroid hormone bioactivity characterized by reduced T3 levels and a shift toward an energetically “hypometabolic” state may lower basal metabolic rate to a degree that compromises thermogenesis, skeletal muscle mass preservation, and physical function in frail or sarcopenic individuals [172,173]. Likewise, recurrent activation of the HPA axis sustains hypercortisolemia, promoting proteolysis, visceral adiposity, immune suppression, mood disturbance, and impaired stress resilience [174]. Perturbations of the RAAS, together with fasting-associated electrolyte shifts can precipitate symptomatic hypotension, orthostasis, or hyponatremia in salt-depleted or polypharmacy-treated patients, particularly those with autonomic dysfunction or advanced chronic kidney disease, even when blood pressure otherwise improves [126,175]. Moreover, unsupervised prolonged fasting can unmask latent adrenal or thyroid insufficiency and precipitate acute metabolic decompensation. However, various high-risk conditions should also be tested in strictly supervised conditions. For example, prolonged fasting was tested in a pilot study including 20 patients with type 1 diabetes and the study demonstrated no ketoacidosis, insulin reduction (24.4 to 7.6 IU), and sustained BMI decrease at 4 months, with quality of life normalization, supporting adaptive benefits under supervision even in high risk phenotypes [176].

Although individual endocrine axes exhibit distinct fasting-induced responses, these adjustments interact within a highly interdependent regulatory network in which changes in one axis can amplify, attenuate, or qualitatively alter responses in another. Shared upstream signals, most notably activation of AMPK and SIRT1 alongside suppression of mTOR activity, coordinate shifts in GH–IGF-1 signaling, thyroid hormone metabolism, adrenal glucocorticoid output, adipokine signaling tone, and RAAS balance. Systemic mediators such as FGF21 and ketone bodies further couple hepatic, adipose, muscular, and hypothalamic pathways, while declining leptin and substrate availability simultaneously shape reproductive and thyrotropic output. Because these pathways converge and modulate one another, the combined endocrine response to prolonged fasting is not merely additive but synergistic and, at times, non-linear. As a result, prolonged fasting can produce emergent physiological states that cannot be predicted from any single axis in isolation. Under favorable conditions, this networked restructuring enhances metabolic efficiency and stress tolerance; however, when fasting exceeds individual physiological reserves or adaptive capacity or occurs in vulnerable phenotypes, the same interlocked mechanisms may potentiate one another in ways that deepen hormonal suppression, dysregulate hemodynamic control, or accelerate catabolic tissue loss. In this dynamic interplay, adaptive and maladaptive responses arise from the same interconnected architecture, with outcomes determined by the degree, duration, and systemic context of endocrine cross-talk. Appreciating this interplay between coordinated adaptive pathways and their potential maladaptive extensions is fundamental to characterizing the physiological landscape of prolonged fasting and to distinguishing it from shorter fasting regimens, where many of these integrated and context-dependent processes remain only partially engaged.

Collectively, these risks argue strongly for individualized, time-limited fasting protocols delivered under medical supervision, with predefined stopping criteria, exclusion of high-risk patients, and structured, stepwise refeeding that prioritizes gradual carbohydrate reintroduction, sufficient high-quality protein intake, and micronutrient repletion to minimize the risk of endocrine or metabolic rebound phenomena.

## 4. Future Research and Perspectives

Translating prolonged fasting from a conceptually compelling physiological intervention into a clinically deployable, safe, and ethically defensible therapeutic strategy requires a coordinated, rigorously structured research agenda. Several pivotal domains warrant prioritization to define the mechanistic foundations, therapeutic potential, and safety boundaries of this practice.

The evidentiary strength across fasting research varies considerably, and a clearer delineation between robust human data and findings derived from small, heterogeneous, or preclinical studies is urgently needed. Much of the current understanding of endocrine and metabolic adaptations relies heavily on animal models or short-term human interventions in narrowly defined populations with limited generalizability, whereas high-quality, well-controlled human trials examining prolonged fasting remain sparse. Without explicit separation of mechanistically informative but still speculative evidence from clinically validated observations, the field risks overstating conclusions and obscuring the true level of certainty, applicability, and translational relevance of existing findings.

A central unresolved question concerns the minimal and optimal fasting durations required to induce hallmark endocrine adaptations. To address this, research priorities should include dose–response RCTs comparing fasting durations from 8 h to ≥4 days, with deep hormonal phenotyping such as pulsatile GH/LH profiling, IGF-1 bioactivity assays, cortisol rhythmicity assessed via 24 h sampling, thyroid deiodinase activity measurements, and RAAS component quantification. Target populations should be stratified by sex (e.g., pre- and postmenopausal women, men with varying androgen levels), age (young adults vs. older individuals ≥65 years), BMI (lean, overweight, obese), and metabolic health (e.g., those with insulin resistance or PCOS). Methodological approaches should integrate multi-omics analyses (transcriptomics, metabolomics, epigenomics) during controlled fasts and targeted mechanistic perturbation studies (e.g., leptin supplementation or GH antagonism). These designs should be coupled with longitudinal follow-up over 12–36 months using validated aging biomarkers (e.g., DNA methylation clocks, glycan age) to define physiological thresholds, characterize inter-axis interactions, and establish long-term safety, thereby enabling phenotype-specific fasting protocols.

Understanding axis-to-axis endocrine cross-talk during nutrient deprivation remains incomplete. Although alterations in GH signaling, thyroid metabolism, the HPA axis, RAAS, and adipokine profiles are well documented, their hierarchical relationships, tissue specificity, and integrated impact on mitochondrial efficiency, circadian timing, immunometabolism, and cellular senescence require clarification. To address this, multi-omics human studies—including transcriptomics, metabolomics, epigenomics, and phosphoproteomics—performed during controlled prolonged fasts are needed. These would determine which endocrine signals operate as primary drivers of healthspan-associated remodeling versus secondary epiphenomena. Mechanistic perturbation studies (e.g., leptin add-back, GH antagonism, glucocorticoid receptor blockade, modulation of deiodinase activity) represent a critical next step in establishing causality.

Pronounced heterogeneity in reproductive axis sensitivity underscores the importance of sex-stratified research. Key gaps include sex-specific fasting thresholds required for metabolic switching; the influence of menstrual cycle, differential susceptibility among premenopausal, perimenopausal, and postmenopausal women to HPG suppression; and the influence of body composition and ovarian reserve. Conversely, potential therapeutic applications in hyperandrogenic states (e.g., PCOS) remain underexplored. Future trials must incorporate high-resolution reproductive hormone profiling with LH/FSH pulsatility, estradiol and progesterone dynamics, testosterone fractions, SHBG, and ovarian/testicular function markers. Equally important is systemic evaluation of androgen-related cardiovascular and metabolic risk modulation in men experiencing intermittent fasting-induced reductions in testosterone.

Marked phenotype-dependent heterogeneity in metabolic flexibility further complicates generalizability. Lean, overweight, and obese individuals differ substantially in mitochondrial function, AMPK signaling, autonomic tone, and hepatic and adipose insulin responsiveness. These differences likely shift the fasting duration required to achieve comparable endocrine adaptations. Rigorous stratification by BMI, visceral adiposity, muscle mass, and metabolic health status, accompanied by objective measures such as DXA, MRI, and indirect calorimetry, is needed to define phenotype-specific fasting protocols and to determine whether repeated fast–refeed cycles can restore metabolic flexibility in metabolically unhealthy obesity.

Refeeding is arguably the most consequential and least standardized phase of prolonged fasting. Key unresolved issues include the optimal macronutrient sequence (protein-first vs. carbohydrate-first), rate of caloric progression, sodium and fluid management, and strategies to prevent post-fast insulin overshoot, edema, electrolyte disturbances, and thyroid hormone rebound. Randomized trials comparing refeeding protocols over 24–72 h, coupled with endocrine endpoints (IGF-1 recovery kinetics, T3/T4 rebound patterns, RAAS normalization, cortisol amplitude restoration), are urgently needed to develop evidence-based and clinically safe refeeding guidelines. Harmonization of refeeding protocols across centers will be essential to enable meta-analysis and reproducible practice standards.

Long-term endocrine safety remains insufficiently characterized. The cumulative effects of repeated prolonged fasts that are often performed autonomously on bone density, fertility, menstrual cyclicity, testosterone levels, adrenal and thyroid function, autonomic nervous system balance, and lean mass preservation remain incompletely understood. Longitudinal studies spanning 12–36 months and incorporating endocrine, autonomic, musculoskeletal, and neurocognitive endpoints, are needed to identify early markers of endocrine fragility that predict intolerance to repeated fasting cycles or predisposition to sustained hormonal dysregulation.

High-risk populations require dedicated safety frameworks. Individuals with diabetes (type 1 or type 2), established cardiovascular disease, chronic kidney disease, osteoporosis or high fracture risk, autonomic dysfunction, adrenal insufficiency, or polypharmacy may derive benefit from fasting yet face substantial risks. Protocols for these groups must emphasize structured medication adjustment algorithms, electrolyte and hemodynamic monitoring, micronutrient supplementation, mandated adequate protein intake during refeeding, and predefined stop criteria. A precision medicine framework leveraging baseline endocrine signatures such as IGF-1 levels, cortisol amplitude, autonomic balance indices, and leptin-to-adiponectin ratios may allow for individualized determination of fasting duration, intensity, and frequency. Case series on prolonged fasting in long COVID patients, demonstrate symptom and metabolic improvements with no severe events, highlighting the need for RCTs with deep endocrine phenotyping to establish protocols for post-viral or inflammatory disorders as models for aging-related conditions [177].

Future studies must extend beyond conventional metabolic endpoints to capture true healthspan effects. Integration of validated biomarkers of biological aging (DNA methylation clocks, glycan age, proteomic and metabolomic aging signatures), measures of organ-specific resilience (vascular reactivity, neurocognitive performance, muscle quality), immune aging metrics, and patient-reported outcomes will be essential. Advanced mediation modeling should delineate which endocrine pathways such as IGF-1 suppression, FGF21 induction, HPA activation, adipokine remodeling, RAAS shifts serve as primary mediators of improved healthspan. Furthermore, lipid-centric trials like the 14-day Buchinger study underscore the value of NMR spectroscopy for lipoprotein subclasses but call for integration with endocrine biomarkers (e.g., insulin/IGF-1) to fully capture healthspan mediators in larger cohorts [178].

Together, these priorities outline a comprehensive, mechanistically grounded, clinically oriented research roadmap. Only through harmonized protocols, deep phenotyping, risk-stratified trial designs, and systematic evaluation of long-term safety will it be possible to determine the true biological consequences of prolonged fasting. Specifically, such efforts are required to establish whether prolonged fasting represents a reversible, beneficial metabolic reprogramming or, in certain contexts, an early step toward endocrine fragility. Addressing these questions will require close integration across endocrinology, metabolism, geroscience, and clinical medicine, ultimately enabling precision-guided and safe implementation of fasting-based interventions with credible potential to enhance human healthspan.

## Data Availability

Not applicable.

## Abbreviations

The following abbreviations are used in this manuscript:

AMPK	AMP-activated protein kinase
Ca^2+^	Calcium ion
CR	Calorie restriction
FGF21	Fibroblast growth factor 21
GH	Growth hormone
GHRH	Growth hormone-releasing hormone
HPA	Hypothalamic–pituitary–adrenal axis
HPG	Hypothalamic–pituitary–gonadal axis
HPS	Hypothalamic–pituitary–somatotropic axis
HPT	Hypothalamic–pituitary–thyroid axis
IF	Intermittent fasting
LH	Luteinizing hormone
MAPK	Mitogen-activated protein kinase
mTOR	Mechanistic target of rapamycin
PGC-1α	Peroxisome proliferator-activated receptor gamma coactivator 1-alpha
PI3K	Phosphoinositide 3-kinase
PKC	Protein kinase C
PLC	Phospholipase C
PPARα	Peroxisome proliferator-activated receptor alpha
RAAS	Renin–angiotensin–aldosterone system
STAT	Signal transducer and activator of transcription
